# ASP-Enzymosomes with *Saccharomyces cerevisiae* Asparaginase II Expressed in *Pichia pastoris*: Formulation Design and In Vitro Studies of a Potential Antileukemic Drug

**DOI:** 10.3390/ijms222011120

**Published:** 2021-10-15

**Authors:** Luciana F. C. Girão, Manuela Colla Carvalheiro, Margarida Ferreira-Silva, Surza L. G. da Rocha, Jonas Perales, M. Bárbara F. Martins, Maria Antonieta Ferrara, Elba P. S. Bon, M. Luísa Corvo

**Affiliations:** 1 Enzyme Technology Laboratory, Department of Biochemistry, Institute of Chemistry, Federal University of Rio de Janeiro, Rio de Janeiro 21941-909, RJ, Brazil; lucygirao@gmail.com; 2 Laboratory of Toxinology, Oswaldo Cruz Institute, Oswaldo Cruz Foundation, Rio de Janeiro 21040-900, RJ, Brazil; surza@ioc.fiocruz.br (S.L.G.d.R.); jperales@ioc.fiocruz.br (J.P.); 3 Instituto de Investigação do Medicamento (iMed.ULisboa), Faculdade de Farmácia, Universidade de Lisboa, Av. Prof. Gama Pinto, 1649-003 Lisbon, Portugal; mcarvalheiro@ff.ulisboa.pt (M.C.C.); ana.m.silva@campus.ul.pt (M.F.-S.); barbarafigueiramartins@edu.ulisboa.pt (M.B.F.M.); 4 Institute of Drug Technology (Farmanguinhos), Oswaldo Cruz Foundation, Rio de Janeiro 21041-250, RJ, Brazil; ferrara.ma@gmail.com

**Keywords:** yeast asparaginase, *ASP3* gene, ASP-enzymosomes, nanoformulations, enzymatic therapy, leukemia treatment

## Abstract

The bacterial enzyme asparaginase is the main treatment option for acute lymphoblastic leukemia. However, it causes side effects, such as immunological reactions, and presents undesirable glutaminase activity. As an alternative, we have been studying asparaginase II from *Saccharomyces cerevisiae*, coded by *ASP3* gene, which was cloned and expressed in *Pichia pastoris.* The recombinant asparaginase (ASP) presented antileukemic activity and a glutaminase activity 100 times lower in comparison to its asparaginase activity. In this work, we describe the development of a delivery system for ASP via its covalent attachment to functionalized polyethylene glycol (PEG) polymer chains in the outer surface of liposomes (ASP-enzymosomes). This new delivery system demonstrated antiproliferative activity against K562 (chronic myeloid leukemia) and Jurkat (acute lymphocytic leukemia) cell lines similar to that of ASP. The antiproliferative response of the ASP-enzymosomes against the Jurkat cells suggests equivalence to that of the free *Escherichia coli* commercial asparaginase (Aginasa^®^). Moreover, the ASP-enzymosomes were stable at 4 °C with no significant loss of activity within 4 days and retained 82% activity up to 37 days. Therefore, ASP-enzymosomes are a promising antileukemic drug.

## 1. Introduction

Therapeutic enzymes have been sourced, from crude plant and animal extracts, as a digestive aid since the end of the 19th century [1]. Since then, their use, production, and formulation have greatly advanced, covering several applications in which highly purified recombinant proteins have been mostly used either in their native form, chemically modified, conjugated to polymers, or nanoformulated to increase stability and effectiveness upon several therapeutic conditions.

Drug delivery systems, such as polymer conjugates, liposomes, and nanoparticles, have demonstrated the capacity to overcome limitations of conventional treatments, reducing immunogenicity, prolonging plasma half-life, and enhancing protein stability. These systems are a very attractive and effective technology [2,3], resulting, for instance, in the successful clinical application of polymer-protein conjugates such as PEGylated enzymes [4,5]. 

Superoxide dismutases (SODs) are a fine example for the advancement of enzyme formulation strategies. Among them, the use of polymers or lipids for SOD conjugation or chemical modification, and the development of SOD-containing liposomal or polymeric nanoparticles for different routes of administration (e.g., parenteral or topical), are included. Moreover, mixed lipid deformable vesicles (e.g., Transfersomes^®^), especially designed for transdermal delivery, were also evaluated [6]. SOD was incorporated either in highly loaded conventional liposomes (SA-liposomes) or long-circulating liposomes (PEG-liposomes) [7]. Besides, SOD was also covalently linked at the distal end of polyethylene glycol (PEG) in the outer membrane of the PEG-liposomes (SOD-enzymosomes), exposing the enzyme on the liposomal surface, displaying enzymatic activity in an intact form without the need for liposomal disruption. This formulation leads to a circulating nanosystem with enzymatic activity with a half-life of about 16 h [8,9]. It is noteworthy that the techniques to perform the liposomal encapsulation of therapeutic enzymes have also greatly evolved with upscaling capacities—a glass-capillary microfluidic technique has been proposed for the preparation of SOD Liposomes [10]. 

The present work focuses on the enzyme asparaginase, used for the treatment of acute lymphoblastic leukemia and non-Hodgkin lymphomas. The proposed mechanism of action, as an anti-leukemia drug, is based on its ability to degrade circulating asparagine, an amino acid that cells of these type of tumors are not able to produce, being dependent on the exogenous source for their metabolic pathways. On the contrary, normal cells are able to produce asparagine, making asparaginase’s antitumor mechanism very selective. The therapeutic approach of this work is to develop a long-circulating system capable of preserving asparaginase’s ability to degrade circulating asparagine with low side effects. Asparaginases derived from the bacteria *Escherichia coli* and *Erwinia chrysanthemi* have been successfully used despite causing adverse effects, such as immunological reactions, presenting an undesirable activity against glutamine, and displaying a short half-life [11,12]. The commercial preparations of *E. coli* asparaginase have been the gold standard and include the purified protein, or the enzyme linked to PEG, a delivery system approved for clinical use—Oncaspar^®^ (pegaspargase). This formulation resulted in an increased plasma half-life from 30 h to 6 days and a significant decrease in adverse effects and immunogenicity [13]. However, its high price impacts the overall cost of treatment. *Erwinia* asparaginase has been used only in cases of immunological reaction to the *E. coli* enzyme. Its half-life is shorter (14 h), and the drug is even more expensive [14]. 

Seeking to further mitigate asparaginase adverse effects, and to improve its pharmacokinetic properties, several studies have been carried out, focusing on the development of asparaginase nanocarrier systems [15,16,17,18]. 

In the pursuit of affordable and better therapeutic options than the use of the bacterial protein, our research group has been studying the *Saccharomyces cerevisiae* asparaginase II, an enzyme encoded by the *ASP3* gene. This gene was cloned and expressed in *Pichia pastoris* heterologous yeast expression system under the control of the *AOX1* gene promoter [19]. The native signal peptide (25 amino acids) plus seven consecutive amino acids were removed and the enzyme yield per dry cell mass for the recombinant *P. pastoris* strain reached 800 U g^−1^, which was 7-fold higher than that obtained for *S. cerevisiae*. High-density cell cultures resulted in a volumetric yield of 85,600 U L^−1^ and global volumetric productivity of 1083 U L^−1^ h^−1^ that are compatible for a biopharmaceutical production. The recombinant periplasmic asparaginase (ASP) was extracted by osmotic shock under alkaline pH, using a 500 mM potassium phosphate, pH 11.5, in the presence of 10 mM cysteine resulting in 100% enzyme recovery [20]. Subsequently, the enzyme was purified to homogeneity by ultrafiltration (50 kDa), molecular exclusion with Superdex 200, and ion-exchange chromatography on a Mono-Q column. After these purification steps, ASP specific activity increased 10.9-fold (204.4 UI mg^−1^) with an activity recovery of 51.3% [21]. The enzyme is glycosylated, non-phosphorylated, and presents a molecular mass under native conditions of 136 kDa, and under reducing conditions of 48.6 kDa and 44.6 kDa, suggesting an oligomeric structure with differences in the glycosylation degree. ASP isoelectric point was 4.5, presenting its maximum activity at 46 °C and pH 7.2. The enzyme was stable up to 45 °C in the pH range 6–10 and retained 92% of its activity at physiological conditions. Moreover, ASP glutaminase activity is 100 times lower in comparison to its asparaginase activity [22]. Structural analysis by circular dichroism and fluorescence demonstrated that the enzyme structure is predominantly made of α-helices with a minor contribution of β-sheets. In vitro studies have demonstrated an antitumoral activity of the recombinant asparaginase against K562 leukemic cells [21].

The present study describes the development of a delivery system for yeast asparaginase, aiming to generate a new antileukemic biopharmaceutical. The innovative formulation was obtained by the covalent attachment of ASP to functionalized PEG polymer chains located at the outer surface of liposomes (ASP-enzymosomes).

## 2. Results

### 2.1. ASP-Enzymosomes and ASP-Liposomes Production and Characterization

**Figure 1:**
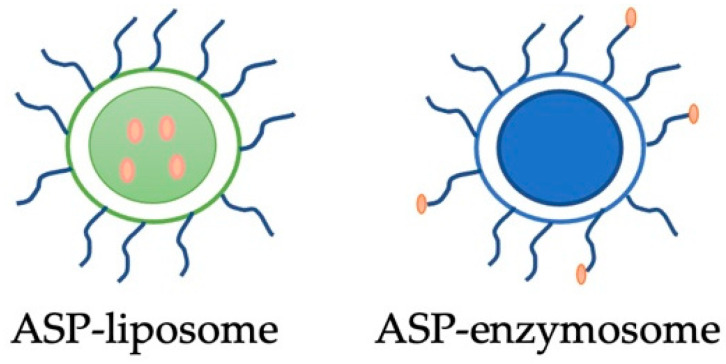
Representation of asparaginase encapsulated into PEG-liposome (ASP-liposome) and attached to PEG molecules at the liposome surface (ASP-enzymosome).

Purified ASP was covalently attached to PEG-liposomes (Figure 1) with the aim to increase plasmatic half-life and reduce potential immunogenic effects. This strategy was successfully applied for the antioxidant enzyme superoxide dismutase [9,23] and is an innovative approach for asparaginase as PEG-asparaginase is the only delivery system currently present in the market for this enzyme. ASP covalently linked to liposomes was named ASP-enzymosomes.

ASP-enzymosomes were prepared by covalent binding between ε-amino groups of lysine residues, in the surface of ASP) and the distal functional (maleimide groups) end of PEG molecules exposed at the surface of the liposomes, which consisted of five steps: (i) ASP thiolation by treatment with N-succinimidyl-S-acetylthioacetate (SATA) reagent to form thiolated ASP (ASP-ATA); (ii) ASP-ATA separation from unreacted SATA by size exclusion chromatography; (iii) ASP-ATA activation with hydroxylamine, resulting in deacetylated ASP-ATA (ASP-AT); (iv) ASP-AT linkage to the PEG-liposomes to form ASP-enzymosome; (v) ASP-enzymosome separation from non-reacted ASP-AT by size exclusion chromatography.

**Figure 2:**
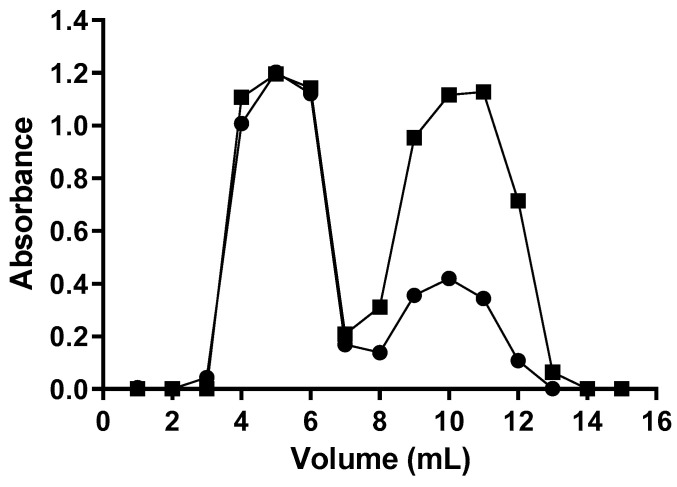
ECONO 10DG column chromatography elution pattern of ASP-ATA and unreacted SATA. (●) absorbance at 280 nm; (■) absorbance at 238 nm. The results presented are from one representative experiment, among the three independent experiments performed.

To allow the covalent binding of ASP to the maleimide groups, exposed at the end of the PEG, linked to the liposomal surface, the ε-amino groups of lysine residues in the surface of ASP needed to be activated, forming ASP-ATA. In order to separate the unreacted SATA reagent from the ASP-ATA, a purification step was necessary. Figure 2 presents size exclusion chromatography profiles of the separation of ASP-ATA from excess of the SATA reagent. The absorbance profile shows a proportional, and matching, increase in absorbances at 280 nm (protein) and 238 nm (SATA) for the fraction corresponding to an elution volume of 4–6 mL, indicating the formation of the ASP-ATA. Excess SATA, which also presents a minor absorption at 280 nm, was eluted in 8–13 mL.

**Figure 3:**
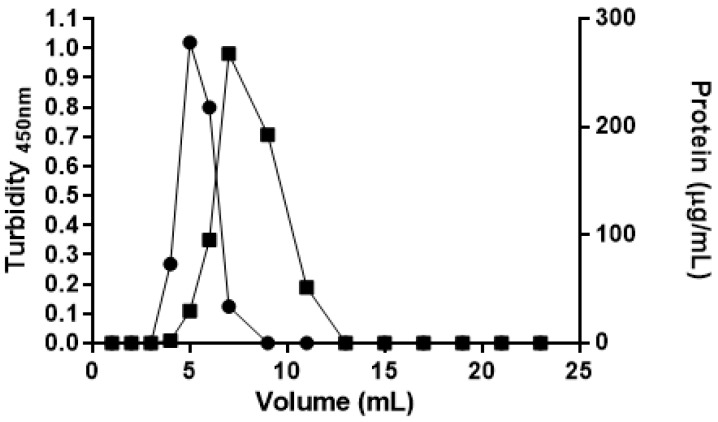
Sephadex G-200 column chromatography elution profile for the purification of ASP-enzymosomes. (●) Liposomes turbidity at 450 nm; (■) Protein concentration measured by Lowry method. The results presented are from one representative experiment, among the three independent experiments performed.

After purification, ASP-ATA was incubated with hydroxylamine and deacetylation solution, to enable the deacylated ASP-AT to link to the maleimide groups present at the end of PEG molecules, thus forming ASP-enzymosomes. To separate the non-covalently attached ASP-AT, a size exclusion chromatography using a Sephadex G-200 column was performed. The chromatographic profiles (turbidity and protein concentration) demonstrates that ASP-enzymosomes were eluted in the void-volume of the column and collected (4 mL to 9 mL fractions), as shown in Figure 3. The free ASP-AT elution was observed in the subsequent fractions, up to 13 mL.

**Figure 4:**
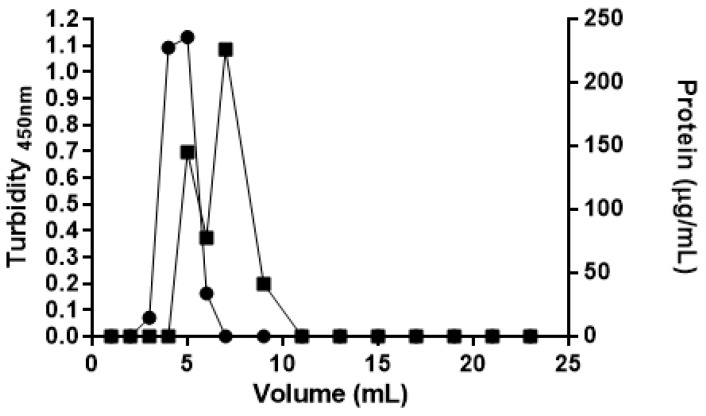
Sephadex G-200 column chromatography elution pattern of ASP-liposomes and non-encapsulated ASP. (●) Liposomes turbidity at 450 nm; (■) Protein concentration measured by Lowry method. The results presented are from one representative experiment, among the three independent experiments performed.

In order to compare ASP-enzymosomes with a formulation previously studied for asparaginase [17], ASP was encapsulated into PEG-liposomes. Afterward, the encapsulated ASP (ASP-liposomes) was separated from the unencapsulated enzyme by size-exclusion chromatography on a Sephadex G-200 column. The chromatographic profile, represented in Figure 4, shows that the ASP-liposomes were eluted in the fraction corresponding to 5 mL, while the enzyme that was not encapsulated or adsorbed to the lipid portion was eluted after the fraction corresponding to 6 mL (in which the lipid concentration is near zero), indicating an efficient purification process.

The results from the characterization of ASP-enzymosomes and ASP-liposomes are summarized in Table 1. It can be observed, for the ASP-enzymosomes, that 93% of ASP-AT was attached to the maleimide groups at the end of PEG, indicating the efficiency of the linking reaction. However, enzyme activity retention was around 48%, suggesting that part of the linked enzyme was inactivated or that ASP modification by the SATA reagent changed the enzyme surface, resulting in a decreased enzyme activity. Therefore, studies of optimization of process variables are necessary to minimize the loss of asparaginase activity on the enzymosomes surface. Regarding ASP-liposomes, the encapsulation percentage of the enzyme was only 16% and was lower than that obtained for *E. coli* asparaginase, which was in the range of 35% [17]. However, asparaginase encapsulated into the liposomes maintained 100% of the specific enzymatic activity, suggesting that this process did not lead to ASP denaturation. No enzymatic activity was detected without previous ASP-liposomal disruption with Triton 4% (*v*/*v*). As the amino acid asparagine is not able to passively cross membranes, the absence of enzymatic activity in intact liposomes indicated that the encapsulated enzyme was located inside the particle, and the enzyme was not adsorbed to its surface.

Considering particle size (Table 1), it is important to notice that, in both formulations, the liposomes presented a similar size, indicating robustness in the process used to prepare these particles. A small increase in mean diameter was observed after the attachment of asparaginase to PEG-liposomes (from 0.143 to 0.165 µm), and the polydispersity index (PdI) remained low. Since the mechanism of action of ASP depends on blood circulation time, the small size obtained is a crucial parameter, because particles larger than 0.2 µm could be trapped and degraded in the liver, reducing the half-life of the formulation [24]. It was also observed that the presence of the enzyme attached to the liposomes slightly increased the negative charge of particles (zeta potential of −8.8 mV). However, due to the presence of PEG on liposomes surface, it is expected that these groups mask ASP, inducing the formation of a hydration sphere, not compromising the long-circulating properties, and avoiding the recognition by the immune system, as observed with SOD-enzymosomes [9].

A conjugation efficiency higher than 90% was observed in the developed ASP-enzymosomes, being similar to that already observed for SOD-enzymosomes [9]. However, ASP-enzymosomes were larger in comparison to the mean size of SOD-enzymosomes, (0.165 µm and 0.120 µm, respectively). As the same conjugation procedure was used for both formulations, the size difference observed between these particles could be attributed to structural and physico-chemical characteristics specific of each enzyme. The use of this innovative methodology, for the formulation of other therapeutical enzymes, could further improve the understanding of this new formulation design.

### 2.2. Formulations Stability

**Figure 5:**
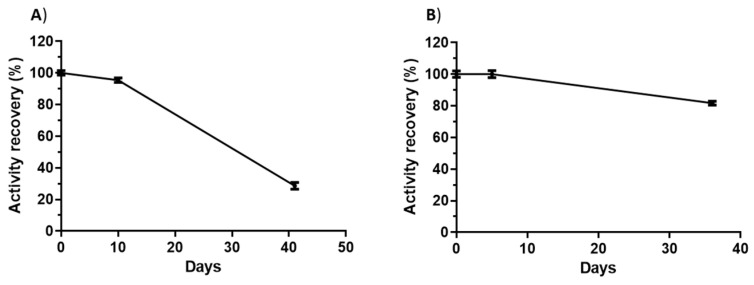
Maintenance of the enzymatic activity at 4 °C: (**A**) ASP-liposomes and (**B**) ASP-enzymosomes. The bars show the standard errors for three replicates from one representative experiment, among the three independent experiments performed.

The stability upon storage of ASP-enzymosomes and ASP-liposomes was studied at 4 °C for 37 days and 42 days, respectively. The following parameters were measured: protein/lipid ratio as an indication of breakdown of the linkage or liposomal disruption and enzymatic activity. The protein/lipid ratio was slightly altered, maintaining 79% and 80% of the initial value for enzymosomes and liposomes, respectively, indicating that formulations have a high stability. Regarding the maintenance of enzymatic activity, as can be observed in Figure 5, ASP-enzymosomes presented higher stability than ASP-liposomes at 4 °C. The ASP-enzymosomes remained stable, with no significant loss of activity for 4 days, and retained approximately 82% of activity after 37 days at 4 °C. These results indicate that this formulation is sufficiently stable—an essential feature for a biopharmaceutical.

The storage stability of ASP-enzymosomes at 4 °C was quite similar to that reported for SOD-enzymosomes, for which the enzymatic activity, after 32 days of storage at 4 °C, was 85% of the initial value [9].

### 2.3. Antiproliferative Activity of the Formulations

To evaluate the activity of ASP-enzymosomes against cancer cells, this formulation was incubated with the cell lines H69 (small cell lung cancer), K562 (human leukemic cell line), and Jurkat (human T lymphocyte cells). Cellular inhibition experiments were also carried out using free ASP, ASP-liposomes, and Aginasa^®^ (commercial *E. coli* asparaginase).

ASP-liposomes that contained encapsulated asparaginase did not inhibit the growth of the studied tumor cells (inhibition < 1%), possibly due to a slow liposome degradation that prevents asparaginase release or to an instability of the released enzyme, with a subsequent loss of catalytic activity.

No growth differences were observed when the H69 cell line was incubated with either ASP-enzymosomes, ASP, Aginasa^®^, or saline solution (inhibition < 1%). This was expected as the H69 cell line presents an active metabolic pathway for the synthesis of asparagine, therefore being independent of external amino acid supply.

**Figure 6:**
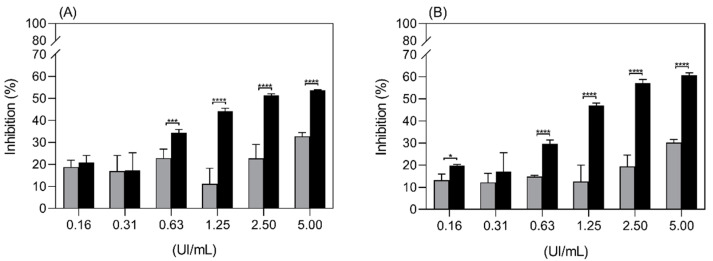
Inhibition of proliferation of the K562 cell line by ASP (grey bars) and Aginasa^®^ (black bars) in 48 h (**A**) and 72 h (**B**). Each point is the mean of six replicates, ±SD, from one representative experiment. * *p*-value < 0.5, *** *p*-value < 0.001 and **** *p*-value < 0.0001.

**Figure 7:**
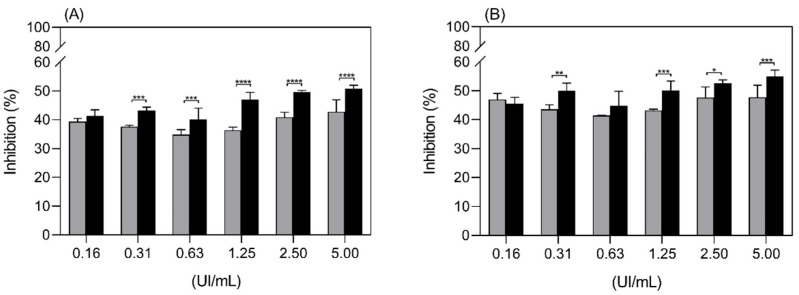
Inhibition of proliferation of the Jurkat cell line by ASP (grey bars) and Aginasa^®^ (black bars) in 48 h (**A**) and 72 h (**B**). Each point is the mean ± SD, from one representative experiment. * *p*-value < 0.5, ** *p*-value < 0.01, *** *p*-value < 0.001 and **** *p*-value < 0.0001.

The cell growth inhibition of the K562 cell line by free ASP and Aginasa^®^ was compared in Figure 6, and a higher efficiency for Aginasa^®^ was demonstrated. This result could be related to the ability of the *E. coli* asparaginase, as opposed to ASP, to also hydrolyze L-glutamine [11]. This effect is a significant feature for some tumor cell lines, particularly the ones less sensitive to L-asparagine depletion, such as K562 [25]. The results of proliferation inhibition assays for Jurkat cell line (Figure 7), which is more prone to the effects of asparaginase depletion than K562 cells, indicated that the antiproliferative response of ASP, despite being somewhat inferior to that of Aginasa^®^ at 48 h, was similar at 72 h, indicating matching antileukemic properties for prolonged periods.

**Figure 8:**
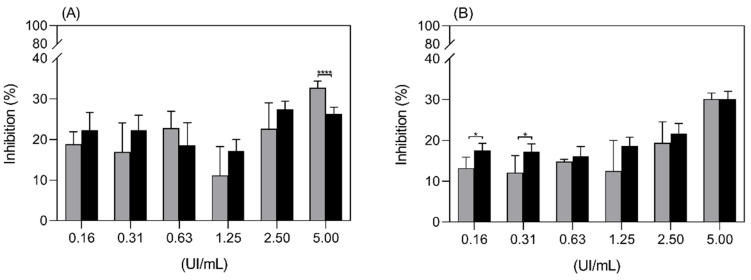
Inhibition of proliferation of the K562 cell line by ASP-enzymosomes (black bars) and ASP (grey bars) at 48 h (**A**) and 72 h (**B**). Each point is the mean of six replicates, ±SD, from one representative experiment. * *p*-value < 0.5 and **** *p*-value < 0.0001.

In Figure 8, the cell growth inhibitory effect of ASP-enzymosomes and ASP towards the K562 cell line was compared, and the observed inhibition pattern is similar.

**Figure 9:**
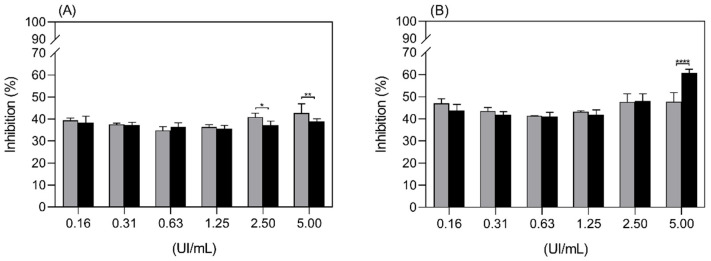
Inhibition of proliferation of the Jurkat cell line by ASP-enzymosomes (black bars) and ASP (grey bars) at 48 h (**A**) and 72 h (**B**). Each point is the mean ± SD, from one representative experiment. * *p*-value < 0.5, ** *p*-value < 0.01 and **** *p*-value < 0.0001.

Results from Figure 9, which compare the inhibition of proliferation of the Jurkat cell line by ASP and ASP-enzymosomes, demonstrate an equivalent antiproliferative effect for both preparations. Thus, the soluble substrate reacted equally with the naked or the stealth enzyme attached to the PEG distal end point. Accordingly, the delivery system was not detrimental to the asparaginase in vitro activity, which is an important feature of the ASP-enzymosome formulation.

The evaluation of the toxic effect of ASP-enzymosomes on normal cells will be performed in the continuation of this study. However, since normal cells present the metabolic pathway for the synthesis of asparagine, and likewise the H69 cell line, it is expected that they will not be negatively affected by the decrease in the amino acid caused by the presence of asparaginase.

The in vitro results, hereby presented and discussed, could be regarded as the first step towards the development of the ASP-enzymosome biopharmaceutical. These findings will be followed by the necessary in vivo studies.

## 3. Materials and Methods

### 3.1. Chemicals

1,2-distearoyl-sn-glycero-3-phosphoethanolamine-N-[maleimide (polyethylene glycol)-2000] (ammonium salt) (maleimide-PEG-DSPE) was purchased from Avanti Polar Lipids, Inc. Egg phosphatidylcholine (Egg-PC) and 1,2-distearoyl-sn-glycero-3-phosphoethanolamine-N-[methoxy (polyethylene glycol)-2000] (PEG-DSPE) were obtained from Lipoid GmbH. N-succinimidyl S-acethylthioacetate (SATA) was from Thermo Scientific Pierce.

### 3.2. Asparaginase Production, Extraction and Purification

Enzyme production was performed in shake flasks using a *P. pastoris* Mut^S^ strain harboring the *S. cerevisiae ASP3* gene, as previously described [19]. Asparaginase was extracted by treating wet cell biomass pellets with 500 mM potassium phosphate solution containing 10 mM cysteine, at pH 11.5 [20].

Asparaginase purification was carried out in four sequential steps. The first one consisted of ultrafiltration in a 50 kDa Amicon system (Millipore^®^) (seven cycles at 4500× *g*, 7 min, at 4 °C, using 50 mM sodium phosphate + 1.2 M ammonium sulfate, pH 7). The enzyme concentrate was then subjected to hydrophobic interaction chromatography in GE Healthcare Phenyl Sepharose CL-4B column (12 mL volume) coupled to an Äkta purifier FPLC system (GE Healthcare), at a flow rate of 1 mL/min. The binding buffer (eluent A) was 50 mM sodium phosphate, 1.2 M (NH4)_2_SO_4_ pH 7.0, whereas the elution buffer (eluent B) was 50 mM sodium phosphate, pH 7.0. Elution was carried out with a linear gradient from 0% to 100% eluent B. Absorbance was measured in 215 and 280 nm, and 1.2 mL fractions were automatically collected using a Frac-950 collector. The pooled active fraction was ultrafiltered in a 10 kDa Amicon system (Millipore^®^) using a 50 mM sodium acetate buffer, pH 5.5 at 4 °C. The fourth purification step consisted of ion-exchange chromatography using a GE Healthcare HiTrap Capto-Q ion-exchange column (0.5 cm × 5 cm, 1 mL volume) coupled to an Äkta purifier FPLC system. One milliliter of sample was applied to the column. Separation was carried out at 1 mL/min in a linear gradient from 0 M NaCl (50 mM sodium acetate buffer, pH 5.5) to 1 M NaCl (50 mM sodium acetate buffer + 1 M NaCl, pH 5.5). Absorbance was measured in 215 and 280 nm, and 1 mL fractions were automatically collected using a Frac-950 collector.

Enzymatic activity of the fractions was measured through the conversion of asparagine and hydroxylamine into aspartohydroxamate [19]. Enzymatically active fractions were analyzed by SDS-PAGE 12% acrylamide gels under reducing conditions [26]. Protein was estimated using a Bio-Rad Protein Assay kit (cat. No. 500-0006), with bovine serum albumin as standard.

### 3.3. Preparation of Long-Circulating Liposomes

Long-circulating liposomes were prepared by encapsulating the purified asparaginase (1 mg mL^−1^) in their aqueous internal space. A mixture of the lipids Egg-PC:Chol:DSPE-PEG (68.25:30.5:1.25 molar ratio) at 32 mM was used. Liposomes were prepared by the dehydration-rehydration method, followed by extrusion, as previously described [9,23,27]. The lipid dispersion containing the enzyme was sequentially extruded through membranes with decreasing pore diameter of 0.6, 0.4, 0.2, and 0.1 μm (Nucleopore^®^ Track-Etched Membranes, Whatman^®^, Florham Park, NJ, USA) using Lipex Thermobarrel extruder. The non-encapsulated protein was separated by ultracentrifugation at 300,000× *g* for 120 min at 15 °C in a Beckman L8-60 M ultracentrifuge followed by Sephadex G-200 size exclusion chromatography using 10 mM sodium citrate buffer + 145 mM NaCl, pH 6.

### 3.4. Preparation of Enzymosomes

Enzymosomes were prepared by covalent binding between ASP and the distal functional end of PEG molecules (Maleimide-PEG-DSPE) exposed at the surface of the liposomes as previously described [9,23].

Liposomes were prepared by mixing the lipids Egg-PC:Chol:Maleimide-PEG-DSPE:PEG-DSPE (molar ratio of 68.25:30.5:0.75:0.50) at 20 mM initial concentration. The liposomes sizing was carried out by sequential extrusion through membrane filters with decreasing pore diameters of 0.6, 0.4, 0.2, and 0.1 μm, resulting in liposomal vesicles.

Prior to liposomes binding, ASP was thiolated by treatment with SATA reagent (SATA:ASP molar ratio of approximately 22:1). The thioacetylated enzyme, ASP-ATA, after separation from unreacted SATA on an Econo-Pac 10DG column (Bio-Rad), was activated with 10 mM citrate + 500 mM hydroxylamine + 1 mM EDTA, pH 6, resulting in ASP-AT. Subsequently, ASP-AT was linked to the liposomes by incubating overnight at room temperature. ASP-enzymosomes were separated from free ASP-AT by Sephadex G-200 molecular exclusion chromatography and concentrated by ultracentrifugation at 300,000× *g* for 120 min at 15 °C in a Beckman LX 90 ultracentrifuge ASP-enzymosomes were stored at 4 °C.

### 3.5. Characterization of Enzymosomes and Liposomes

The encapsulated or linked protein was measured by the Lowry method [28]. Phospholipid was quantified by the Rouser method [29]. The enzymatic activity was measured before and after the disruption of the liposomes with Triton 4% (*v*/*v*). The mean size and polydispersity of formulations were measured by dynamic light scattering (DLS) in a Zetasizer NanoS (Malvern Instruments^®^, Malvern, UK). The surface charge of particles (zeta potential) was measured by laser-doppler anemometry in a Zetasizer NanoZ (Malvern Instruments^®^, Malvern, UK).

### 3.6. Enzymosomes and Liposomes Stability Assays

ASP-liposomes and ASP enzymosomes stability were evaluated at 4 °C, during 37 and 42 days, respectively. The following parameters were characterized: enzyme and phospholipid amount in liposomal form and retention of enzymatic activity.

### 3.7. Antiproliferative Activity in Leukemic Cells

The antiproliferative activity of the formulations (enzymosomes and liposomes) was studied in K562 (myelogenous leukemia cell line, immortalized cell line K562 derived from a CML patient was obtained from ATCC, reference no. CCL-243 (Manassas, VA, USA)), Jurkat (T lymphocyte cell line, cells (clone E6-1) were obtained from ATCC), and H69 (small cell lung cancer, human classical SCLC cell line NCI-H69 was obtained from ATCC, reference no. HTB-119) cell lines. Free *S. cerevisiae* asparaginase expressed in *P. pastoris* (ASP), commercial E. coli asparaginase (Aginasa^®^, Kyowa Hakko Kirin Co., Ltd., Tokyo, Japan), and saline solution were used as controls.

The assays were performed according to the MTS protocol [30]. Briefly, cells that were maintained in the exponential growth phase were seeded in 96-well plates, at 2.5 × 10^3^ cells per well. Afterward, cells were treated with ASP-liposomes, ASP-enzymosomes, or controls (0.08 to 10 IU/mL) and incubated at 37 °C and 5% CO_2_. After 48 h or 72 h, the MTS reagent (3-(4,5-dimethylthiazol-2-yl)-5-(3-carboxymethoxyphenyl)-2-(4-sulfophenyl)-2H-tetrazolium) was added, and the plates were incubated at 37 °C for 2 h, after which the absorbances were read at 490 nm and 630 nm wavelength.

### 3.8. Statistical Analysis

A one-way analysis of variance (ANOVA), followed by Sidak’s multiple comparison test, was performed to determine whether the observed differences were statistically significant. Statistical software Graph Pad Prism (version 8.0; Graph Pad software, San Diego, CA, USA) was used for all analyses. Significance levels are described in figure legends.

## 4. Conclusions

A new delivery system (ASP-enzymosomes) was successfully developed for the therapeutic enzyme asparaginase (ASP), via the enzyme covalent attachment, to functionalized polyethylene glycol (PEG) polymer chains in the outer surface of liposomes. Upon incubation at 4 °C, the formulation remained fully stable for 4 days and retained 82% enzymatic activity within 37 days, showing a stability compatible to its use as a biopharmaceutical. The ASP-enzymosomes demonstrated in vitro antiproliferative activity against K562 and Jurkat cell lines that was equivalent to that of ASP. The antiproliferative response of the ASP-enzymosomes against the Jurkat cells suggests equivalence to that of the free *E. coli* commercial asparaginase (Aginasa^®^). The continuation of the studies for ASP-enzymosomes production and use could potentially result in a valuable biopharmaceutic alternative for acute lymphoblastic leukemia treatment.

## 5. Patents

A patent application was filed for the asparaginase formulation reported in this paper (BR102018069598, Registration institution: INPI—National Institute of Industrial Property, Brazil. INPI Deposit: 09/25/2018; WIPO deposit: 09/25/2019).

## Figures and Tables

**Table 1:** Characterization of ASP-enzymosomes and ASP-liposomes.

Properties	ASP-Enzymosomes *	ASP-Liposomes **
Final Protein/Lipid Ratio (µg/µmol)	70.8 ± 5.3	5.9
Binding/Encapsulation of ASP-AT to liposomes (%) ***	93.5 ± 6.5	16.2
Retention of ASP activity (%)	47.6 ± 5.0	100.0
Lipid yield (%)	60.3 ± 9.6	75.3
Zeta Potential (mV) at pH 6.0 ****	−8.8 ± 1.8	−2.0
Size (µm) (PdI) ****	0.165 ± 0.005 (0.122 ± 0.023)	0.140 (0.078)

* Values represent the mean and standard deviation of three independent experiments. ** Values in accordance with values previously obtained for enzymes [9]. *** Calculated in relation to the theoretical value before the incubation of ASP-AT with liposomes. **** Liposomes before conjugation with ASP: zeta potential = −3.1 ± 0.5 mV; size 0.143 ± 0.007 µm (PdI = 0.055).

## Data Availability

The data presented in this study are available on request from the corresponding author.

## Abbreviations

ASP	purified *S. cerevisiae* asparaginase II expressed in *P. pastoris*
ASP-ATA	thiolated ASP
ASP-AT	deacetylated ASP-ATA
DLS	dynamic light scattering
Egg-PC	egg phosphatidylcholine
maleimide-PEG-DSPE	1,2-distearoyl-sn-glycero-3-phosphoethanolamine-N-[maleimide (PEG)-2000] (ammonium salt)
PC	phosphatidylcholine
PdI	polydispersity index
PEG	polyethylene glycol
PEG-DSPE	1,2-distearoyl-sn-glycero-3-phosphoethanolamine-N-[methoxy (PEG)-2000]
SATA	N-succinimidyl S-acethylthioacetate
SOD	superoxide dismutase
